# Computed Tomography Radiomics for Residual Positron Emission Tomography-Computed Tomography Uptake in Lymph Nodes after Treatment

**DOI:** 10.3390/cancers12123564

**Published:** 2020-11-28

**Authors:** Chu Hyun Kim, Hyunjin Park, Ho Yun Lee, Joong Hyun Ahn, Seung Hak Lee, Insuk Sohn, Joon Young Choi, Hong Kwan Kim

**Affiliations:** 1 Department of Radiology and Center for Imaging Science, Samsung Medical Center, Sungkyunkwan University School of Medicine, Seoul 06351, Korea; ch27.kim@samsung.com; 2 School of Electronic and Electrical Engineering, Sungkyunkwan University, Suwon 16419, Korea; hyunjinp@skku.edu; 3 Center for Neuroscience Imaging Research, Institute for Basic Science, Suwon 16419, Korea; 4 Department of Health Sciences and Technology, SAIHST, Sungkyunkwan University, Seoul 06355, Korea; 5 Statistics and Data Center, Research Institute for Future Medicine, Samsung Medical Center, Seoul 135-710, Korea; jhguy.ahn@samsung.com (J.H.A.); insuks@gmail.com (I.S.); 6 Department of Electrical and Computer Engineering, Sungkyunkwan University, Suwon 16419, Korea; victor87822@gmail.com; 7 Department of Nuclear Medicine, Samsung Medical Center, Sungkyunkwan University School of Medicine, Seoul 06351, Korea; jynm.choi@samsung.com; 8 Department of Thoracic & Cardiovascular Surgery, Samsung Medical Center, Sungkyunkwan University School of Medicine, Seoul 06351, Korea; hkts.kim@samsung.com

**Keywords:** lung cancer diagnosis, lymph nodes, imaging

## Abstract

**Simple Summary:**

In this study we explored the predictive ability of radiomics in non-small cell lung cancer patients, and reported the complementary role of radiomics in predicting the treatment response of the lymph nodes. Radiomics analysis is a cutting-edge technology for the noninvasive assessment of tumor biology, which converts medical images into mineable high-dimensional data. Our method is cost-effective with no need for additional studies, and moreover, we used an easily reproducible study method that can be applicable in further studies using radiomics in oncology.

**Abstract:**

Although a substantial decrease in 2-[fluorine-18]fluoro-2-deoxy-d-glucose (FDG) uptake on positron emission tomography-computed tomography (PET-CT) indicates a promising metabolic response to treatment, predicting the pathologic status of lymph nodes (LN) remains challenging. We investigated the potential of a CT radiomics approach to predict the pathologic complete response of LNs showing residual uptake after neoadjuvant concurrent chemoradiotherapy (NeoCCRT) in patients with non-small cell lung cancer (NSCLC). Two hundred and thirty-seven patients who underwent NeoCCRT for stage IIIa NSCLC were included. Two hundred fifty-two CT radiomics features were extracted from LNs showing remaining positive FDG uptake upon restaging PET-CT. A multivariable logistic regression analysis of radiomics features and clinicopathologic characteristics was used to develop a prediction model. Of the 237 patients, 135 patients (185 nodes) met our inclusion criteria. Eighty-seven LNs were proven to be malignant (47.0%, 87/185). Upon multivariable analysis, metastatic LNs were significantly prevalent in females and patients with adenocarcinoma (odds ratio (OR) = 2.02, 95% confidence interval (CI) = 0.88–4.62 and OR = 0.39, 95% CI = 0.19–0.77 each). Metastatic LNs also had a larger maximal 3D diameter and higher cluster tendency (OR = 9.92, 95% CI = 3.15–31.17 and OR = 2.36, 95% CI = 1.22–4.55 each). The predictive model for metastasis showed a discrimination performance with an area under the receiver operating characteristic curve of 0.728 (95% CI = 0.654–0.801, *p* value < 0.001). The radiomics approach allows for the noninvasive detection of metastases in LNs with residual FDG uptake after the treatment of NSCLC patients.

## 1. Introduction

Although lung cancer remains the leading cause of worldwide cancer mortality [1], the use of molecular targeted agents [2] and the expansion of the role of immunotherapy using checkpoint inhibitors have significantly advanced treatment [3]. The accurate and precise assessment of treatment response in lung cancer patients has correspondingly become increasingly important [4].

It is difficult to use computed tomography (CT) to assess lymph node (LN) metastasis, in contrast to other metastases, because the LN itself has its own size, and Positron emission tomography-computed tomography (PET-CT) is therefore commonly used to evaluate the response of LNs after treatment [5]. Although a substantial decrease in 2-[fluorine-18] fluoro-2-deoxy-d-glucose (FDG) uptake is promising, false negative and false positive values of 25% and 33%, respectively, have been reported when predicting the pathologic complete response of LNs [6]. These high false negative and positive values indicate that PET-CT images alone are not suitable for making a final decision and additional invasive pathologic confirmation is recommended.

Radiomics is a quantitative, noninvasive method of obtaining information embedded within conventional CT images [7]. Multiple studies using a radiomics approach in oncology have reported favorable results. As the radiomics approach allows the better characterization of tumors, more precise prognostic assessment [8], and an improved prediction of drug resistance [9,10], it may be used as a helpful tool for evaluating the treatment response of lymph nodes also.

Many studies have reported the efficacy of evaluating treatment response in lung cancer [11,12,13]; however, no study evaluating the treatment response of lymph nodes using a radiomics approach has been published in the literature.

Here, we investigated the potential of a CT radiomics approach to predict the pathologic complete response of LNs with residual uptake upon PET-CT after neoadjuvant concurrent chemoradiotherapy (NeoCCRT) in patients with stage IIIa/N2 non-small cell lung cancer (NSCLC).

## 2. Results

### 2.1. Patient Characteristics

The study comprised a total of 135 patients. The mean age was 61 years (range, 53–68) and most of the patients were male (*n* = 106, 78.5%). More than half of the patients were adenocarcinoma (*n* = 84, 62.2%). Clinical T stage was cT1 in 30 patients (22%), cT2 in 90 (67%) and cT3 in 15 (9.6%). All patients had N2 disease. The pathologic T stage was ypT1 in 29 (21%), ypT2 in 85 (63%), ypT3 in 59 (15%), and ypT4 in 17 (3%). The pathologic N stage was ypN0 in 65 patients (44%), ypN1 in 18 (13%), and ypN2 in 52 (38%).

**Figure 1:**
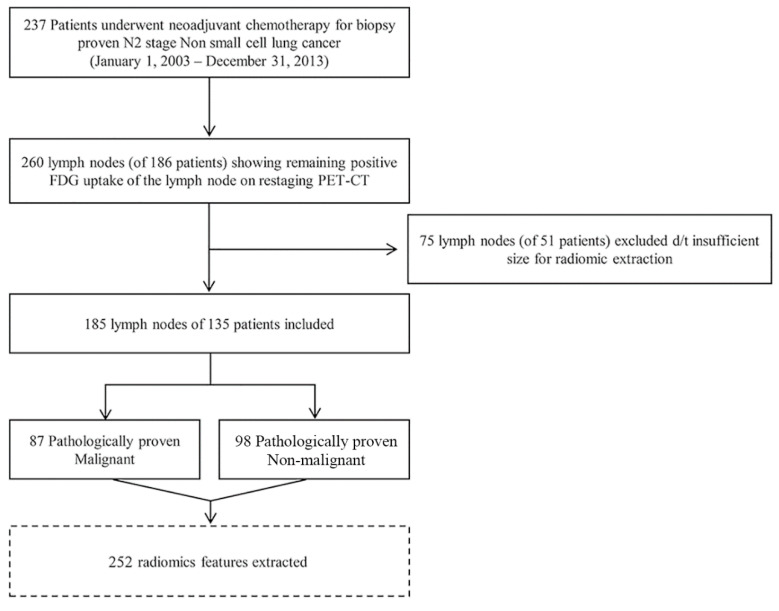
Patient enrollment. In total, 237 patients were identified who underwent neoadjuvant concurrent chemoradiotherapy and consecutive complete curative resection from the thoracic surgical database of our institution.

In total, 185 LNs in 135 patients were selected in the study. Of the 185 LNs in 135 patients, 87 LNs of 70 patients were pathologically proven to be malignant and the remaining 98 LNs in the other 65 patients were pathologically proven to be non-malignant (Figure 1). The characteristics of patients with pathology-negative LNs and pathology-positive LNs are shown in Table 1. There were no significant differences in mean age, tumor size, primary tumor SUVmax, number of lymph nodes involved in PET-CT or pT stage between the two groups. The percentage of females was significantly higher in the pathology-positive group (28.6% (20/70) versus 13.8% in the pathology-negative group (9/65)). Percentage of adenocarcinoma was significantly higher in the pathology-positive group, at 74.3% (52/70), than the pathology-negative group, at 49.2% (32/65).

Among them, 260 lymph nodes (LN) from 186 patients showed remaining positive 2-[fluorine-18]fluoro-2-deoxy-d-glucose uptake upon restaging positron emission tomography-computed tomography, and were selected. In total, 75 LNs from 51 patients were excluded due to insufficient size. Finally, we enrolled 185 LNs of 135 patients as the study group.

### 2.2. Analyses of Clinical/Radiomics Variables for Predicting Malignant LNs

In the univariate analyses of demographic and pathologic parameters (Table 2), sex (*p* = 0.028) and cell type (*p* = 0.004) were significantly associated with malignant LNs. Female sex and adenocarcinoma cell type were calculated risk factors. Age and T staging were not risk factors.

A total of 252 features were extracted from CT images. Four radiomics features, namely ‘volume’ and ‘maximal 3D diameter’ from the shape and physical-based category, ‘cluster tendency’ from the GLCM-based category, and ‘offset deviation’ from the sigmoid function based-category, were selected by the LASSO logistic regression model (Figure S1). Selected radiomics features showed predictive efficacy, with an AUC of 0.6779 (95% confidence interval (CI), 0.599–0.756). Of the four variables, ‘volume’ from the shape and physical-based category and ‘offset deviation’ from the sigmoid function based-category were excluded due to their small coefficient value, which proved to be unstable in 10-fold cross-validation. As a result, two radiomics features were chosen for the construction of the radiomics signature, as follows: ‘maximal 3D diameter’ from the shape and physical-based category, and ‘cluster tendency’ from the GLCM-based category.

**Figure 2:**
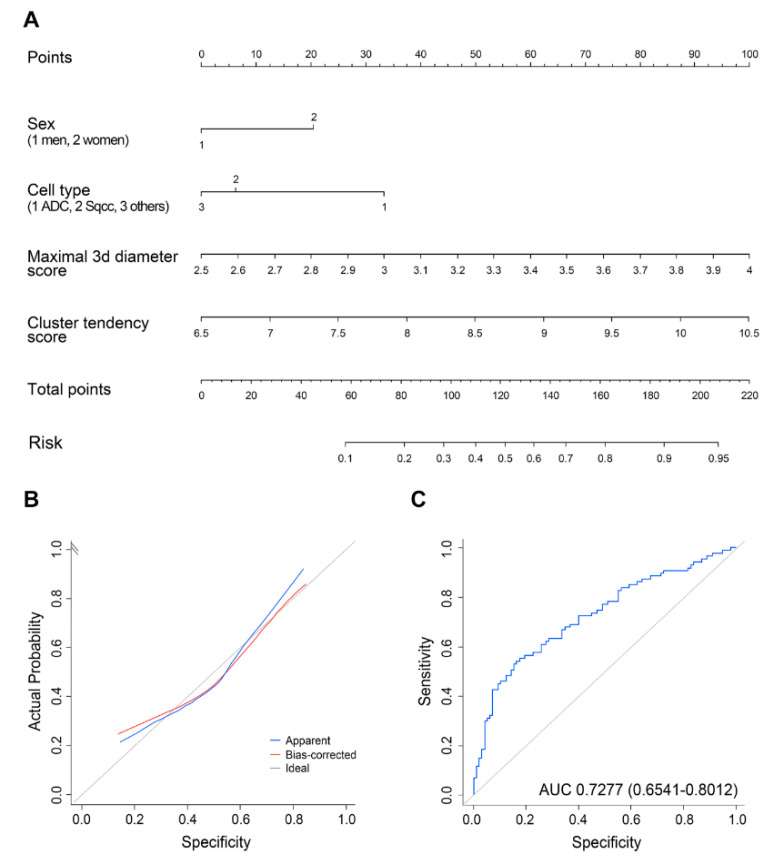
Radiomics nomogram. (**A**) Nomogram was constructed on the basis of the prediction model of a metastatic lymph node. (**B**) Calibration curves of the nomogram. (**C**) Receiver operating characteristic curves from 10-fold cross-validation.

**Figure 3:**
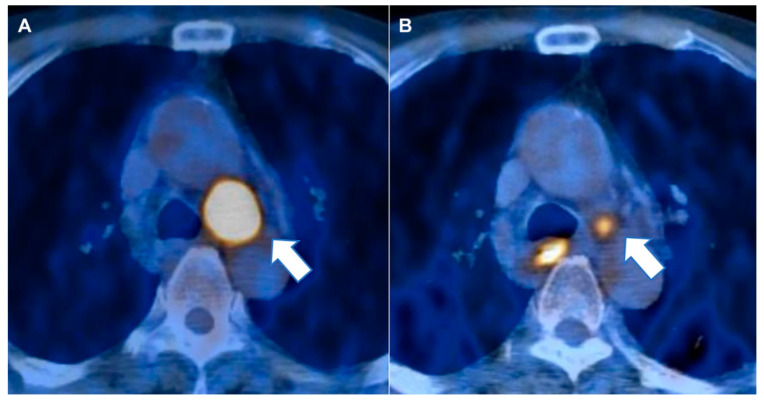
A 79-year-old man diagnosed with T1bN2M0 stage squamous lung cancer. Upon positron emission tomography-computed tomography (PET-CT) taken before neoadjuvant concurrent chemoradiotherapy (NeoCCRT) (**A**), a primary mass in the left lower lobe of lung was found with metastasis to the ipsilateral paratracheal lymph node (arrow) showing increased 2-[fluorine-18]fluoro-2-deoxy-d-glucose (FDG) uptake. Upon PET-CT taken after NeoCCRT (**B**), an interval decrease in size and FDG uptake of paratracheal lymph node (arrow) are seen, but a higher FDG uptake than normal in mediastinal blood pooling remained, suggesting the greater likelihood of residual malignant tissue. However, the calculated risk for malignancy in the predictive nomogram was 34.22% (Sex 1; Cell type 2; Maximal 3d diameter score 3.17; Cluster tendency score 8.58; Total points 102.73). Pathologic diagnosis for the lymph node proved benign with no residual malignancy.

The results of multivariable logistic regression analysis demonstrated that the two selected radiomics features, adenocarcinoma, and female sex were independent predictors of metastatic LN (Table 3). Therefore, a radiomics nomogram was constructed with the selected radiomics features, sex, and cell type as predictors of metastatic LNs (Figure 2A). This model showed a favorable AUC of 0.728 (95% confidence interval (CI), 0.654–0.801) and good calibration (Figure 2B,C). Figure 3 shows a representative case showing the efficacy of the predictive nomogram.

## 3. Discussion

The accurate staging of LNs is essential, because it affects the selection of treatment plans and the survival rate of cancer patients. Although various methods are currently used to evaluate LN status, assessing LN status remains challenging.

Radiomics analysis, which converts medical images into mineable high-dimensional data, is a promising method for the noninvasive assessment of tumors. The approach has been adopted in various fields within oncology. Radiomics data, in conjunction with other information, have been shown to be correlated with clinical outcomes data, and have been used to support evidence-based clinical decision-making [14].

In this study, we explored the ability of radiomics to predict LN metastases in treated NSCLC patients. To analyze the CT radiomics feature, we used the CT component of PET-CT. This approach is simple and cost-effective, and makes the most of the combined information obtained from a single PET/CT study, with no further requirement for an additional CT study.

Various methods are currently used to evaluate lymph nodes. When assessing lymph nodes by CT, the size of the LN is widely used to infer the possible presence of metastasis; an enlarged lymph node (usually one with a short axis of ≥10 mm) is considered abnormal [15]. However, preoperative mediastinal lymph node staging in NSCLC by CT was shown to have a low sensitivity, ranging between 0.33 and 0.75, and a specificity of 0.66–0.90 [16]. It is therefore not surprising that the accuracy of the CT scans for assessing treatment response is low. Other studies reported that when restaging mediastinal nodes in NSCLC patients after treatment, CT had a sensitivity of 41–67% and a specificity of 67–75% [6,17]. However, the additional diagnostic benefit of MRI is questionable, and MRI has a limited ability to accurately stage nodal involvement [18]. Several studies have reported that PET-CT is more accurate than CT and MRI for pathological staging. However, false negative and false positive rates of 20–25% and 25–33%, respectively, with regard to predicting the pathologic complete response of lymph nodes, have been reported [6,19]. This indicates that the PET-CT findings should not be used to make a final decision, and invasive nodal biopsies are required.

Upon reviewing the pathology of the patients who had a negative result for pathology with false positive PET-CT results, 38.5% (25 of 65) showed fibrosis with central necrotic lymph nodes suggesting tumoral necrosis. In total, 32.3% (21 of 65 patients) were thought to have hyperplastic reactive lymph nodes due to the underlying background of obstructive pneumonia. In total, 15.4% (10 of 65 patients) had anthracofibrotic lymph nodes. The most interesting thing during this review was that 10.8% (7 of 65 patients) showed superimposed tuberculosis lymphadenitis, the proportion of which is quite a substantial and reasonable result given that it is endemic in our country. Additionally, 1.5% (1 of 65) were related to sarcoidosis, and the residual 1.5% (1 of 65) were related to actinomycosis infection.

In the current study, we found that the combination of radiomics features and clinical–pathologic risk factors in a radiomics nomogram had a favorable AUC of 0.728. By comparison, the sensitivity, specificity, and negative predictive value of conventional PET/CT parameters in our patient cohort were 45.6%, 85.0% and 79.3%, respectively, and they showed a receiver operating characteristic curve of 0.653 (95% CI = 0.571–0.735). Additionally, a receiver operating characteristic curve of 0.627 (95% CI = 0.545–0.71) was calculated when using only nodal size for predicting metastatic LN in the CT.

The identified radiomics features predictive of metastatic LN were maximal 3D diameter, a shape and physical-based feature, and cluster tendency, which is a GLCM-based feature. Maximal 3D diameter was calculated as the largest distance between voxels in the ROI. In our data, the mean maximal 3D diameter of pathology-positive lymph nodes was 27.94 ± 9.33 mm, whereas the mean maximal 3D diameter of pathology-negative lymph nodes was 23.97 ± 8.14 mm. Although it is well known that larger lymph nodes are more likely to be malignant [15], the fact that only a few millimeters separate the LN sizes in these two groups makes it difficult for radiologists to discriminate between pathology-positive and -negative lymph nodes by assessing size on conventional CT scans. The other selected radiomics feature, cluster tendency, is the number of potential clusters present in the gray level co-occurrence matrix, and this reflects the heterogeneity of the lesion. Tissue heterogeneity is a hallmark of malignancy and poor prognosis [8,20]. The selected features have well-grounded biological relevance, and we believe they can predict malignant lymph nodes because they reflect the abnormal proliferation and alteration of tissue architecture in malignancies. In sum, a radiomics approach can be effective for diagnosing small lesions that cannot be perceived by the radiologist’s naked eye.

Adenocarcinoma was also identified as a risk factor for residual metastatic lymph nodes in the present patient cohort. This result is consistent with that reported in previous studies; namely, that adenocarcinoma is one of the known risk factors for occult mediastinal metastases in NSCLCs [21,22].

Female gender was another independent risk factor. A previous study reported that the occult mediastinal node involvement of NSCLC occurred more frequently in females [23], but further studies are needed to confirm these results.

Our study had several limitations. First, it was a retrospective study with potential selection bias. Furthermore, the image acquisition and reconstruction parameters were heterogeneous due to the retrospective nature of this study. However, this variability may ultimately be considered an advantage as radiomics features are likely to be extracted across multiple institutions with different imaging protocols. Second, we did not perform an external validation using an independent cohort. Due to our small sample size, this analysis was based on a derivation cohort only, with the testing set being drawn from the same group as the training set. Instead, we performed a 10-fold cross-validation to generate possible validation data. Third, there is a potential limitation of LASSO, in that it is sensitive to outliers. Since the range of radiomics variables is large, the radiomics variables were first converted to a maximum value 1 and a minimum value 0, and then the variable was selected using the LASSO method. This method is less sensitive to outliers than raw data. Lastly, radiomics itself has limitations. Radiomics is a high-dimensional analysis approach whereby hundreds of features are computed. To properly model the radiomics features, we need more samples than conventional imaging studies, wherein only a few (most likely less than ten) features are considered [13,24]. Another limitation is the reproducibility of the radiomics features. The features are mathematically defined by utilizing minute details in the raw data, and thus small changes in raw intensity, possibly coming from variability in the image acquisition settings, might affect the reproducibility of the features [25]. This, in turn, could lead to alterations in the radiomics features.

## 4. Materials and Methods

### 4.1. Patients

This study was performed in accordance with the Declaration of Helsinki. This single institution retrospective study was approved by the Institutional Review Board of Samsung Medical Center with waiver of informed consent (Institutional Review Board, file number 2018-09-004). Between November 2003 and December 2013, we identified 237 patients with N2 stage NSCLC (based on the TNM stage 7th edition) who underwent NeoCCRT and consecutive complete curative resection. The exclusion criteria were severe cardiopulmonary impairment precluding surgery, double primary lung cancer, and carcinoid lung cancer.

The histopathologic confirmation of N2 mediastinal nodal metastasis was performed through surgical (mediastinoscopy, chamberlain incision, or thoracoscopy) or non-surgical methods (endobronchial ultrasound–transbronchial needle aspiration). Our institution has adopted a multidisciplinary approach to manage patients with lung cancer, and trimodality therapy including NeoCCRT followed by surgical resection has been the primary recommendation for patients with N2 NSCLC. Thoracic radiotherapy was administered to patients with a total dose of 45 Gy delivered over 5 weeks from 1997 to 2009, or a dose of 44 Gy delivered over 4.5 weeks in later periods. The TRT target volume included the known gross and clinical disease, and adequate peripheral margins. Regarding the chemotherapy regimens, intravenous cisplatin and oral etoposide were used from May 1997 to March 2001, and thereafter, intravenous paclitaxel or docetaxel was administered in combination with cisplatin or carboplatin. The first dose of chemotherapy was delivered on the first day of TRT. Within 3 or 4 weeks following the completion of neoadjuvant therapy, restaging. Unless the restaging workup uncovered evidence of progressive disease, surgical resection was planned for 4–6 weeks following the completion of neoadjuvant therapy. The patients who progressed on neoadjuvant chemotherapy did not undergo surgery and continued on radiotherapy. Surgical resection was performed with curative intent, and it consisted of the complete resection of the primary tumor and systematic dissection of the hilar and mediastinal lymph nodes. For resection of the primary tumor, lobectomy, bi-lobectomy and pneumonectomy were performed. In patients who underwent lobectomy (81%, 194 of 237), a mean number of 16.87 (range 5–46) lymph nodes were dissected. A mean number of 18.43 lymph nodes (range 7–50) for bi-lobectomy (8.9%. 21 of 237) and a mean number of 17.86 lymph nodes (range 2–52) for pneumonectomy (9.3%, 22 of 237) were dissected each. The mean number of dissected lymph nodes in all patients was 17.06 (range, 2–44). A mean of 5.57 (range 2–9) lymph node stations were resected during the surgery.

Among 237 patients, 260 LNs of 186 patients showed remaining positive FDG uptake upon restaging PET-CT. Seventy-five LNs from 51 patients were excluded because the LNs were small and were covered by fewer than three sequential axial slices in CT, which is not sufficient for radiomics analysis (all had a longest diameter less than 3.75 mm). Thus, we enrolled 135 patients as the final study group (Figure 1).

### 4.2. Image Analysis and Region of Interest Segmentation

Both the PET and CT components in PET-CT were used in evaluating LNs. Integrated PET-CT was performed as follows. Blood glucose levels were checked below 150 mg/dL, before the injection of 370 MBq (10 mCi) of FDG. Patient rested for over 45 min before scanning. The dedicated PET/CT device consisted of an eight-slice CT scanner (Light-Speed Plus; GE Healthcare, Milwaukee, WI, USA) and a PET scanner (Advance NXi; GE Healthcare, Milwaukee, WI, USA). A non-enhanced CT scan was obtained using a continuous spiral technique (140 kVp and 80 mA adjusted on the basis of the patient’s weight, and section width of 5 mm). After performing the CT, an emission scan from thigh to head was performed in the same transverse field of view.

A nuclear medicine physician with 11 years of experience in PET-CT interpretation who was blinded to the clinical and pathologic data evaluated all PET images. For semiquantitative analysis of FDG uptake in lymph nodes, regions of interest (ROIs) were placed over the most intense area of FDG accumulation. When FDG uptake could not be assessed on the PET component images of PET-CT, an ROI was drawn in a presumed location by taking into consideration CT component images of PET-CT. The FDG uptake within the ROIs was calculated as SUVmax. LNs with SUVmax values greater than background were considered to be metastatic [26].

**Figure 4:**
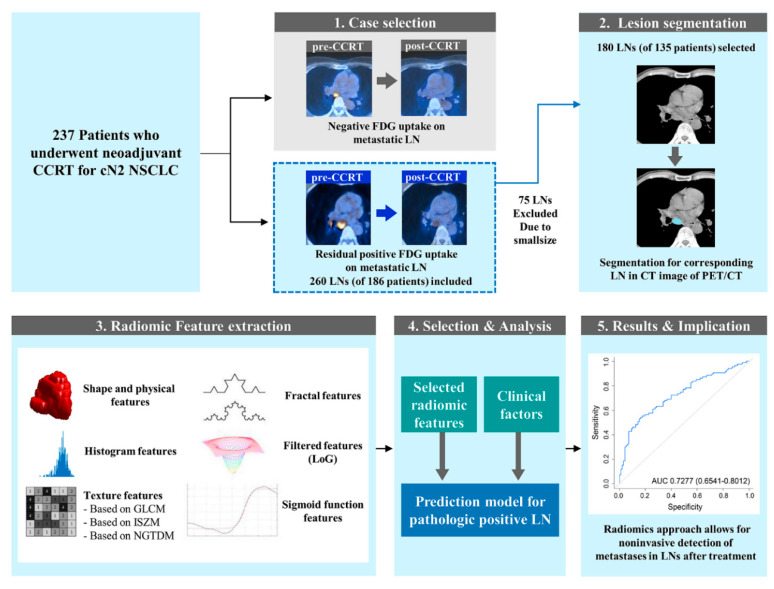
Overall processing steps. In the case selection step, the presumed metastatic lymph nodes upon pre-concurrent chemoradiotherapy (CCRT) positron emission tomography-computed tomography (PET-CT) with abnormal residual 2-[fluorine-18]fluoro-2-deoxy-d-glucose uptake in post-CCRT PET-CT were selected. The region of interest of the whole lymph node was independently segmented in the CT component of PET-CT. In this study, a total of 252 features were extracted for the ROI from the raw imaging data. The features belonged to one of the following six categories: (1) shape and physical-based, (2) histogram-based, (3) texture-based, (4) fractal-based, (5) filter-based, and (6) sigmoid function-based. The final step of the study was to construct a prediction model for pathologic positive lymph nodes incorporating selected radiomics features and clinical factors.

Figure 4 provides an overview of the imaging analysis steps and procedures. First, patients with a residual abnormal FDG uptake and presumed metastatic LNs in post-CCRT PET-CT images were selected. We then independently segmented ROIs in the selected LNs in the CT component of PET-CT using a semi-automated process [27], using the mediastinal window setting (window width, 400 Hounsfield Units [HU]; window level, 20 HU). Radiomics feature extraction of the segmented LNs was then performed.

### 4.3. CT Radiomics Feature Extraction

In this study, a total of 252 radiomics features were extracted using a combination of open source software and in-house MATLAB code [28,29]. Features unavailable from the open source software were implemented locally. Features were divided into six categories: (1) shape and physical-based, (2) histogram-based, (3) texture-based, (4) fractal-based, (5) filter-based, (6) and sigmoid function-based. Shape and physical-based features reflect the morphology of an ROI. They are commonly used by radiologists to describe lesions, but in radiomics analysis, they refer to three-dimensional quantification with computer assistance [14]. Three shape and physical-based features were computed from the slice where the largest tumor was located. Other features were 3D features. As for software, histogram-, texture- and shape-based (3D) features (*n* = 165) were extracted using PyRadiomics version 1.3.0 [30]. Log-, fractal-, 2D shape-, and sigmoid-based features (*n* = 87) were not available in PyRadiomics, thus the feature extraction was performed using in-house code implemented in MATLAB (Natick, MA, USA, version 2016b). A detailed explanation of the CT radiomics technique and selected radiomics features are listed in Appendix A and Tables S1 and S2.

### 4.4. Statistical Analyses

Continuous variables for two patient groups (i.e., pathologic positive LNs group and pathologic negative lymph nodes group) were compared using the *t* test or Mann–Whitney test, and categorical data were compared using the χ^2^ or McNemar test. Two-tailed *p* values < 0.05 were considered to be statistically significant.

Univariate logistic analysis was performed to determine the predictors of malignant LNs. The variables explored included demographics and histopathologic characteristics. For the radiomics features, we used the least absolute shrinkage and selection operator (LASSO) logistic regression algorithm to select LN status-related features among the 252 imaging features we extracted. For the multivariable analysis, all demographic and pathologic characteristics that demonstrated statistical significance in the univariate analysis, and radiomics features selected from LASSO logistic regression, were included. Multivariable logistic regression with stepwise selection was used to fit a model to predict malignant LNs. A radiomics nomogram was constructed on the basis of the prediction model. Calibration of the nomogram was assessed with a calibration curve. To evaluate the predictive performance of the prediction model, a 10-fold cross-validation procedure was used. Predicted probability values for all patients were calculated by 10-fold cross-validation and combined. A single receiver operating characteristic (ROC) curve was drawn and the area under the curve (AUC) was calculated. All statistical calculations were performed using SAS (version 9.4; SAS Institute, Cary, NC, USA) and R (version 3.3.1; Vienna, Austria; http://www.R-project.org/ [31]) software.

## 5. Conclusions

In conclusion, the radiomics approach described here had fair diagnostic yields in the noninvasive detection of LN metastases after neoadjuvant chemoradiotherapy. It might be able to predict the status of the LNs in NSCLC patients that show residual PET uptake after treatment.

## Figures and Tables

**Table 1:** Demographic and clinical characteristics (*n* = 135).

Characteristics	Patients with Pathologic Negative LNs (*n* = 65)	Patients with Pathologic Positive LNs (*n* = 70)	*p*-Value
Mean age ± SD (years)	61.83 ± 7.063	60.66 ± 7.549	0.480
Gender			0.037
Male	56 (86.2%)	50 (71.4%)	
Female	9 (13.8%)	20 (28.6%)	
Tumor size, mm (mean ± SD)	49.8 ± 23.2	47.2 ± 21.3	0.675
SUVmax of tumor (mean ± SD)	13.6 ± 4.1	12.3 ± 4.8	0.127
Number of lymph nodes involved in PET-CT (median)	2 (1–6)	2 (1–5)	0.130
pT stage			0.872
1a	6 (9.2%)	7 (10.0%)	
1b	9 (13.8%)	7 (10.0%)	
2a	28 (43.1%)	36 (51.4%)	
2b	11 (16.9%)	10 (14.3%)	
3	11 (16.9%)	10 (14.3%)	
Histopathology			0.011
Adenocarcinoma	32 (49.2%)	52 (74.3%)	
Squamous cell carcinoma	30 (46.2%)	16 (22.9%)	
Non-small cell lung cancer *	3 (4.6%)	2 (2.9%)	

* large cell carcinoma, giant cell carcinoma, poorly differentiated carcinoma.

**Table 2:** Univariate analysis and pathologic variables for predicting malignant lymph nodes.

Category	Variable	Reference	OR	95% CI	*p*-Value
Demographic factors	Sex	Male	2.286	1.095–4.770	0.028
	Age		0.258	0.043–1.552	0.139
Pathologic factors	pT	pT1	0.667	0.237–1.873	0.442
	Cell type	Adenocarcinoma	0.406	0.219–0.755	0.004

OR = odds ratio; CI = confidence interval.

**Table 3:** Multivariable analysis to select predictive factors for the nomogram.

Variable	Reference	OR	95% CI	*p*-Value
Sex	Male	2.02	0.88–4.62	0.096
Cell type	Adenocarcinoma	0.39	0.19–0.77	0.0073
Maximal 3D diameter		9.80	3.14–30.61	<0.0001
Cluster tendency		2.36	1.23–4.57	0.0099

OR = odds ratio; CI = confidence interval.

## Data Availability

The datasets generated during and/or analyzed during the current study are available from the corresponding author on reasonable request.

## Supplementary Materials

The following are available online at https://www.mdpi.com/2072-6694/12/12/3564/s1, Figure S1: Selection of radiomics features using the least absolute shrinkage and selection operator (LASSO) logistic regression model, (**A**) LASSO coefficient analysis of 161 radiomics features. (**B**) Coefficient plotted against the log (λ) sequence. Four nonzero coefficients (indicated by a vertical line in the plot) were selected. (**C**) Predictive accuracy of the radiomics signature, Table S1: Radiomics features based on histogram, shape and size, texture, fractal, filters, and sigmoid functions, Table S2: Definitions of extracted radiomics features.

## Appendix A

The radiomics process starts with image acquisition. Following image acquisition, the tumor region of interest (ROI) is defined for analysis (i.e., tumor segmentation), and generally includes the entire tumor area. Next, from the defined ROI, a variety of radiomics features are extracted via software programs. The currently available radiomics features can be classified into multiple categories. Representative categories include morphological, histogram-based, texture, and airway-related radiomics features.

Finally, feature selection, model development, and performance testing are some of the most critical and demanding steps of a radiomics study. Feature selection may either be performed simultaneously with model development as a built-in procedure or may be done separately. Although numerous features can be extracted from a single tumor, the numerous available features need to be reduced to a practical number. Thus, through feature selection and model development, the most useful and prognostic radiomics features are summarized for clinical application. Several classifiers can be used for the model development depending on the number of cases, features and characteristics. Lastly, testing the performance of the selected radiomics features is crucial to popularize radiomics in clinical research [32].

Histogram-based features are first order statistics derived from the intensity distribution of voxels in a given ROI. Texture features including gray level co-occurrence matrix-based (GLCM) features, intensity size zone matrix (ISZM)-based features, and neighborhood gray tone difference matrix (NGTDM) features were considered [14]. GLCM features are based on the intensity values of a neighborhood instead of a single voxel. Similarities and dissimilarities in voxel intensity patterns within a neighborhood can be quantified. ISZM-based features also quantify texture, but they assume that the ROI can be further divided into subregions of uniform intensity and variable size. ISZ quantifies the number of subregions and how often certain subregions occur within the ROI. NGTDM-based features measure the difference between a gray value and the mean gray value of their neighborhoods. These are also texture measures. We computed five NGTDM features [33].

For fractal features, the fractal dimension was calculated using box-counting and fractal signature dissimilarity was calculated using the blanket method. Lacunarity represents the texture or distribution of different sized gaps within an image and was computed using the box-counting method [34,35].

To extract filtered-based features, we adopted the 3D Laplacian of a Gaussian filter [13,36]. We computed features at various scales denoted by the sigma value (σ) from 0.5 to 3.5 in 0.5 increments.

For sigmoid function-based features, the sigmoid function was used to fit density change along a sampling line drawn orthogonal to the tumor surface. We considered orthogonal lines of length 3 mm, 5 mm, or 7 mm inside and outside the tumor [13].
