# Supplementary material for: Computed Tomography Radiomics for Residual Positron Emission Tomography-Computed Tomography Uptake in Lymph Nodes after Treatment
Source: Cancers (Basel). 2020 Nov 28;12(12):3564. doi: 10.3390/cancers12123564 (PMC7761511; doi:10.3390/cancers12123564)
Supplement: Supplementary file 1 [file cancers-12-03564-s001.pdf]

Supplementary Materials: Computed Tomography Radiomics for Residual Positron Emission Tomography-Computed Tomography Uptake in Lymph Nodes after Treatment

Chu Hyun Kim, Hyunjin Park, Ho Yun Lee, Joong Hyun Ahn, Seung-hak Lee, Insuk Sohn, Joon Young Choi and Hong Kwan Kim

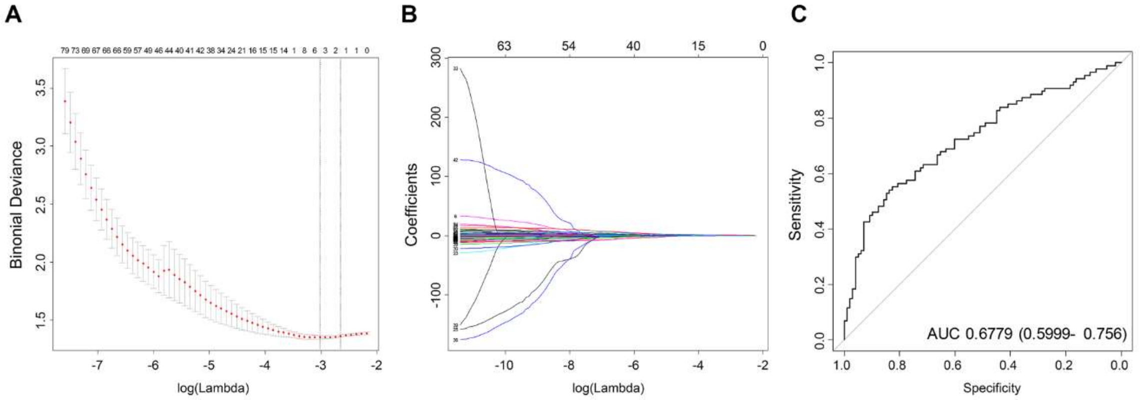

Figure S1. Selection of radiomics features using the least absolute shrinkage and selection operator (LASSO) logistic regression model. (**A**) LASSO coefficient analysis of 161 radiomics features. (**B**) Coefficient plotted against the log (λ) sequence. Four nonzero coefficients (indicated by a vertical line in the plot) were selected. (**C**) Predictive accuracy of the radiomics signature.

Table S1. Radiomics features based on histogram, shape and size, texture, fractal, filters, and sigmoid functions.

Histogram Features		Shape and Size Features	Texture Features		
**Based on Whole Pixels**	**Based on Positive Pixels**	**Based on 2D, 3D Images**	**Based on GLCM**	**Based on ISZ**	**Based on NGTDM**
Maximum*	Mean	Compactness	Auto correlation**	Size zone variance	Busyness
Minimum*	Standard variation	Surface area	Cluster tendency**	Intensive variance	Coarseness
Median*	Variance	Convexity	Maximum probability**		Complexity
Mean*	Maximum	Sphericity	Contrast**		Contrast
Variance*	Median	Spherical disproportion	Difference entropy**		Strength
Standard variation*	Minimum	Maximum 3D diameter	Dissimilarity**		
Energy	Interquartile range	Surface-to-volume ratio	Energy**		
Skewness*	Range	Volume	Entropy**		
Kurtosis*	Root mean square	Density	Homogeneity**		

Root mean square	Skewness	Mass	Informational measure of correlation**
Inter quartile range	Energy	Roundness factor	Variance**
Range	Entropy	Eccentricity	
Percentile 2.5%, 25%, 50%, 75%, 97.5%	Kurtosis	Solidity	
Entropy*	Uniformity		
Uniformity			
Mean value of positive pixels			
Uniformity of positive pixels			

**Fractal Features**		**Filtered Features (LoG***)**	**Sigmoid Function Features**
**Based on the Box-Counting Method**	**Based on the Blanket Method**	**σ = 0.5–3.5**	**3,5,7 mm**
Dimension	Fractal signature dissimilarity	Mean	Amplitude mean
Lacunarity		Max	Amplitude standard deviation
		Min	Slope mean
		Median	Slope standard deviation
		Standard deviation	Offset mean
		Skewness	Offset standard deviation
		Kurtosis	
		Uniformity	
		Entropy	

ISZ, intensity variance and size zone variance value; GLCM, gray-level co-occurrence matrix; NGTDM, neighborhood gray tone difference matrix; LoG, Laplacian of Gaussian. *These features were calculated from the whole, inner 2/3, and outer 1/3 of the ROI. Difference (delta) between inner and outer ROIs was computed. **These features were calculated from the setting of * plus sub-sampled ROIs. ***Sigma values for LoG features were computed for σ = 0.5–3.5 in 0.5 increments.

Table S2. Definitions of extracted radiomics features.

Category	Parameter	Formula	Description
Histogram-based features [1]	Max, Min	$\text{Max} = Max(X(i))$ or $\text{Min} = Min(X(i))$ where X denotes the 3D image matrix with N voxels.	Measures maximum or minimum intensity value of a histogram
	Median	$\text{Median} = \frac{X(i)}{2}$ where X denotes the 3D image matrix	Measures median intensity value of a histogram
	Mean	$\text{Mean} = \frac{1}{N}\sum_{i}^{N} X(i)$ where X denotes the 3D image matrix with N voxels.	Measures mean intensity value of a histogram

Variance	$\text{Variance} = \frac{1}{N-1}\sum_{i=1}^{N} (X(i) - \bar{x})^2$	Measures squared distances of each value of a histogram from the mean
Standard deviation	$\text{Std} = \left(\frac{1}{N-1}\sum_{i=1}^{N} (X(i) - \bar{x})^2\right)^{1/2}$ where X denotes the 3D image matrix with N voxels.	Measures amount of variation of a histogram.
Energy	$\text{Energy} = \sum_{i}^{N} X(i)^2$ where X denotes the 3D image matrix with N voxels.	Measures squared magnitude value of a histogram
Skewness	$\text{Skewness} = \frac{E(x-\mu)^3}{\sigma^3}$ where μ is the mean of x, σ is the standard deviation of x, and E is the expectation operator.	Measures asymmetry of a histogram.
Kurtosis	$\text{Kurtosis} = \frac{E(x-\mu)^4}{\sigma^4}$ where μ is the mean of x, σ is the standard deviation of x, and E is the expectation operator.	Measures "peakedness" of a histogram (flatness of histogram)
Root mean square (RMS)	$\text{RMS} = \sqrt{\frac{1}{N}\sum_{n=1}^{N} \lvert X_n \rvert^2}$ where X denotes the 3D image matrix with N voxels.	Measures the square root of the mean of the squares of the values of the histogram. This feature is another measure of the magnitude of a histogram
Interquartile range	$\text{IQR} = Q_3 - Q_1$ where Q_3 denote the 3rd quartile of the histogram, and Q_1 denotes the 1 st quartile of the histogram	Measure of variability, based on dividing a histogram into quartiles
Range	$\text{Range} = \text{range}(X(i))$	Measures difference between the highest and lowest voxel values of a histogram
Percentile	$\text{Percentile} = \left(\frac{n^{th}\ percentile}{100}\right) X(i)$	Measures intensity value at the 2.5th, 25th ,50th ,75th, and 97.5th percentiles on the histogram
Entropy	$\text{Entropy} = -\sum_{i=1}^{N_l} P(i)\log_2 P(i)$ where P denotes the first-order histogram with N_l discrete intensity levels.	Measures irregularity of a histogram.
Uniformity	$\text{Uniformity} = \sum_{i=1}^{N_l} P(i)^2$ where P denotes the first-order histogram with N_l discrete intensity levels.	Measures uniformity of a histogram.
Mean value of positive pixels (MPP)	$\text{MPP} = \frac{1}{N_+}\sum_{i}^{N} X(i)$ where N_+ denotes the total number of positive gray level pixels in $X(i)$	Measures average positive histogram value.
Uniformity value of positive pixels (UPP)	$\text{UPP} = \sum_{i=1}^{N_l} \lvert P(i) \rvert^2$	Measures uniformity of a positive histogram value.

		where P denotes the first-order histogram with N_l discrete intensity levels.	
Shape- and physical-based features [1,2]	Compactness	$\text{Compactness} = \frac{V}{\sqrt{\pi}A^{\frac{3}{2}}}$ where V denotes the volume and A denotes the surface area of the volume of interest (VOI)	Quantifies how close an object is to the smoothest shape, the circle
	Surface area	$\text{SA} = \sum_{i=1}^{N} \frac{1}{2} \lvert a_i b_i \times a_i c_i \rvert$ where N is the total number of triangles (covered surface area), and a, b, c are edge vectors	Surface area of the ROI
	Convexity	$\text{Convexity} = \frac{V}{V'}$ where V denotes tumor volume and V' denotes convex hull volume	Measures the ratio of the ROI volume contained within the tumor to the calculated convex hull volume
	Sphericity	$\text{Sphericity} = \frac{\pi^{\frac{1}{3}} \times (6\text{V})^{\frac{2}{3}}}{\text{A}}$ where A denotes area and V denotes tumor volume	Measures the roundness of the ROI
	Spherical disproportion	$\text{Spherical disproportion} = \frac{A}{4\pi R^2}$ where R is the radius of a sphere with the same volume as the tumor	Ratio of the surface area of the ROI to the surface area of a sphere with the same volume as the ROI
	Maximum 3D diameter	See description in the next column	Measures the maximum 3D ROI diameter. This was measured as the largest pairwise Euclidean distance between surface voxels of the ROI
	Surface-to-volume ratio (SVR)	$\text{SVR} = \frac{A}{V}$ where A is area and V is volume	Surface-to-volume ratio in ROI
	Volume	$\text{Volume} = \text{R} * \text{number of voxels}$ where R denotes the 3D image resolution	Volume of the tumor (ROI)
	Mass	$\text{Mass} = \text{V} * \text{D}$ where V denotes the tumor volume, and D denotes the tumor density	Mass of the tumor (ROI)
	Density	$\text{Density} = \frac{M}{V}$ where V denotes the tumor volume and M denotes the tumor mass	Density of the tumor (ROI)
	Roundness factor (2D)	$\text{Roundness factor} = \frac{4\pi \cdot Area}{Perimeter^2}$	Measure of circularity of a ROI
	Eccentricity (2D)	$\text{Eccentricity} = \text{c}/\text{a}$ where c is the distance from the center to a focus and a is the distance from that focus to a vertex	Measure of how close the tumor shape is to a circle
	Solidity (2D)	$\text{Solidity} = \frac{Area}{Convex\ area}$	Measure of convexity of a ROI on the 2D image
GLCM-based features [1]	Auto correlation	$\text{Autocorrelation} = \sum_{i=1}^{N_g} \sum_{j=1}^{N_g} ij\boldsymbol{P}(i,j)$	Measure of the magnitude of the fineness and coarseness of texture

	Cluster tendency	$\text{Cluster tendency} = \sum_{i=1}^{N_g}\sum_{j=1}^{N_g}[i + j - \mu_x - \mu_y]^2 \mathbf{P}(i,j)$	Measure of the homogeneity of the GLCM
	Maximum probability	$\text{Maximum probability} = \max\{P(i,j)\}$	Measure of the maximum value of the GLCM matrix
	Contrast	$\text{Contrast} = \sum_{i=1}^{N_g}\sum_{j=1}^{N_g}\lvert i - j\rvert^2 \mathbf{P}(i,j)$	Measures of the local intensity variation of the GLCM
	Difference entropy	$\text{Difference entropy} = \sum_{i=0}^{N_g-1} \mathbf{P}_{x-y}(i)\log_2[P_{x-y}(i)]$	Measure of the entropy of the processed GLCM matrix Px-y
	Dissimilarity	$\text{Dissimilarity} = \sum_{i=1}^{N_g}\sum_{j=1}^{N_g}\lvert i - j\rvert \mathbf{P}(i,j)$	Measure of the difference in each element of the gray level
	Energy	$\text{Energy} = \sum_{i=1}^{N_g}\sum_{j=1}^{N_g}[\mathbf{P}(i,j)]^2$	Measure of the homogeneity of the GLCM
	Entropy	$\text{Entropy} = -\sum_{i=1}^{N_g}\sum_{j=1}^{N_g}\mathbf{P}(i,j)\log_2[\mathbf{P}(i,j)]$	Measure of the irregularity of the gray level.
	Homogeneity	$\text{Homogeneity} = \sum_{i=1}^{N_g}\sum_{j=1}^{N_g}\frac{\mathbf{P}(i,j)}{1 + \lvert i - j\rvert}$	Measure of the closeness of the gray level.
	Informational measure of correlation	$\text{IMC} = \text{HXY} - \frac{\text{HXY1}}{\max\{HX, HY\}}$	Secondary measure of homogeneity
	Variance	$\text{Variance} = \sum_{i=1}^{N_g}\sum_{j=1}^{N_g}(i - \mu_x)^2 P(i,j)$	Measure of the dispersion of parameter values around the mean of the combinations of reference and neighborhood pixels

where $\mathbf{P}(i,j)$ is the gray level co-occurrence matrix for $(\delta = 1, \alpha = 0)$,
N_g is the number of discrete intensity value in the image,
N is the number of voxels in the ROI,
μ is the mean of $\mathbf{P}(i,j)$,
$p_x(i) = \sum_{j=1}^{N_g}\mathbf{P}(i,j)$ is the marginal row probabilities,
$p_y(i) = \sum_{i=1}^{N_g}\mathbf{P}(i,j)$ is the marginal column probability,
μ_x is the expected value of marginal row probability,
μ_y is the expected value of the marginal column probability,
σ_x is the standard deviation of p_x,
σ_y is the standard deviation of p_y,
$p_{x+y}(k) = \sum_{i=1}^{N_g}\sum_{j=1}^{N_g}\mathbf{P}(i,j)$, $i + j = k, k = 2,3,\ldots,2N_g$,
$p_{x-y}(k) = \sum_{i=1}^{N_g}\sum_{j=1}^{N_g}\mathbf{P}(i,j)$, $\lvert i - j\rvert = k, k = 0,1,\ldots,N_g - 1$,
$HX = -\sum_{i=1}^{N_g}\mathbf{P}_x(i)\log_2[p_x(i)]$ is the entropy of $\mathbf{P}_x$,
$HY = -\sum_{i=1}^{N_g}\mathbf{P}_y(i)\log_2[p_y(i)]$ is the entropy of $\mathbf{P}_y$,
$HXY = -\sum_{i=1}^{N_g}\sum_{j=1}^{N_g}\mathbf{P}(i,j)\log_2[\mathbf{P}(i,j)]$ is the entropy of $\mathbf{P}(i,j)$

$$HXY1 = -\sum_{i=1}^{N_g}\sum_{j=1}^{N_g}\mathbf{P}(i,j)\log(p_x(i)p_y(j))$$

ISZ-based features [3]	Size-zone variability	$\text{Size zone variability} = \frac{1}{\Theta}\sum_{m=1}^{M}\left[\sum_{n=1}^{N}\boldsymbol{P}(m,n)\right]^2$	Variability in the size of the ROI
	Intensity variability	$\text{Intensity variability} = \frac{1}{\Theta}\sum_{n=1}^{N}\left[\sum_{m=1}^{M}\boldsymbol{P}(m,n)\right]^2$	Variability in the intensity of the ROI

		where $\boldsymbol{P}(m,n)$ is the intensity size zone matrix<br>Θ represents the number of homogeneous areas in the tumor,<br>M is the number of distinct intensity values,<br>N is the size of homogeneous area in the matrix $\boldsymbol{P}(m,n)$	
NGTDM-based features [4,5]	Busyness	$\text{Busyness} = \sum_{i=1}^{L} p_i s(i) / \sum_{i=1}^{L}\sum_{j=1}^{L}(ip_i - jp_j)$	Measure of the spatial rate of gray-level change
	Coarseness	$\text{Coarseness} = \left[\sum_{i=1}^{L} p_i s(i)\right]^{-1}$	Measure of edge density
	Complexity	$\text{Complexity} = \sum_{i=1}^{L}\sum_{j=1}^{L}\{(\lvert i-j\rvert) / (n^2(p_i + p_j))\}\{P_i s(i) + p_j s(j)\}$	Measure of the amount of information in an ROI (gray-level intensities, number of sharp edges)
	Contrast	$\text{Contrast} = \frac{1}{N_g(N_g-1)}\sum_{i=1}^{L}\sum_{j=1}^{L} p_i p_j (i-j)^2 \cdot \frac{1}{n^2}\sum_{i=1}^{L} s(i)$	Measure of local variations and spread of matrix values
	Strength	$\text{Strength} = \sum_{i=1}^{L}\sum_{j=1}^{L}(p_i + p_j)(i-j)^2 / \sum_{i=1}^{L} s(i)$	
		where p_i is the probability of occurrence of a gray level value, $s(i)$ is the NGTDM, N_g is the total number of different gray levels in the ROI, and L is the number of possible gray levels	
Filter-based features [2] (LoG)	Mean	$\text{Mean} = \frac{1}{\text{N}}\sum_{\text{i}}^{\text{N}} \text{G(i)}$<br>where G denotes the filtered 3D image matrix with N voxels.	Measurement of the mean of the ROI image processed by the LoG filter
	Max	$\text{Max} = Max(G(i))$<br>where G denotes the filtered 3D image matrix with N voxels.	Measurement of the maximum intensity value of the ROI image processed by the LoG filter
	Min	$\text{Min} = Min(G(i))$<br>where G denotes the filtered 3D image matrix with N voxels.	Measurement of the minimum intensity value of the ROI image processed by the LoG filter
	Median	$\text{Median} = \frac{\text{G(i)}}{2}$<br>where G denotes the filtered 3D image matrix	Measurement of the median intensity value of the ROI image processed by the LoG filter
	Standard deviation (Std)	$\text{Std} = \left(\frac{1}{\text{N}-1}\sum_{\text{i}=1}^{\text{N}} (\text{G(i)} - \bar{G})^2\right)^{1/2}$<br>where G denotes the filtered 3D image matrix with N voxels.	Measurement of the standard deviation of the ROI image processed by the LoG filter
	Skewness	$\text{Skewness} = \frac{E(G-\mu)^3}{\sigma^3}$<br>where μ is the mean of G, σ is the standard deviation of G, and E is the expectation operator.	Measurement of the skewness of the ROI image processed by the LoG filter

	Kurtosis	$\text{Kurtosis} = \frac{E(G-\mu)^4}{\sigma^4}$ where μ is the mean of G, σ is the standard deviation of G, and E is the expectation operator.	Measurement of kurtosis of the ROI image processed by the LoG filter
	Uniformity	$\text{Uniformity} = \sum_{i=1}^{N_l} P(i)^2$ where P denotes the first-order histogram with N_l discrete intensity levels.	Measurement of the uniformity of the ROI image processed by the LoG filter
	Entropy	$\text{Entropy} = -\sum_{i=1}^{N_l} P(i)\log_2 P(i)$ where P denotes the first-order histogram with N_l discrete intensity levels.	Measurement of entropy of the ROI image processed by the LoG filter
		$G(x,y,z,\sigma) = I(x,y,z) * \frac{1}{\sigma(\sqrt{2\pi})^3} e^{-\frac{x^2+y^2+z^2}{2\sigma^2}}$ σ = 0.5 − 3.5, 0.5 increments, where I(x,y,z) is the image and * denotes convolution	
Fractal-based features [6,7]	Lacunarity (box-counting method)	See description in the next column	Measure of the texture or distribution of gaps within an image
	Dimension (box-counting method)	$\text{Fractal dimension} = \lim_{r\to 0}\frac{\log(N_r)}{\log(1/r)}$ where N_r is the number of voxels and r is the different side lengths	Fractal dimension that quantifies morphological complexity and provides information on self-similarity properties
	Fractal signature dissimilarity (blanket method)	See description in the next column	Measure of tumor heterogeneity
Sigmoid function-based features [2]	Amplitude mean	See description in the next column	Mean of the amplitude values of all samplings lines
	Amplitude standard deviation		Standard deviation of the amplitude values of all sampling lines
	Slope mean		Mean of the slope values of all sampling lines
	Slope standard deviation		Standard deviation of the slope values of all sampling lines
	Offset mean		Mean of the offset values of all sampling lines
	Offset standard deviation		Standard deviation of the offset values of all sampling lines
		$\text{Sigmoid(x)} = \frac{A}{e^{B\cdot x}+1} + \text{C}$ where A is the amplitude, B is the slope of the curve, and C is the offset of the curve	

ISZ, intensity variance and size zone variance value; GLCM, gray-level co-occurrence matrix; NGTDM, neighborhood gray tone difference matrix; LoG, Laplacian of Gaussian.

References

1. Aerts, H.J.; Velazquez, E.R.; Leijenaar, R.T.; Parmar, C.; Grossmann, P.; Carvalho, S.; Bussink, J.; Monshouwer, R.; Haibe-Kains, B.; Rietveld, D.; et al. Decoding tumour phenotype by noninvasive imaging using a quantitative radiomics approach. *Nat. Commun.* **2014**, *5*, 4006.
2. Aerts, H.J.; Grossmann, P.; Tan, Y.; Oxnard, G.R.; Rizvi, N.; Schwartz, L.H.; Zhao, B. Defining a Radiomic Response Phenotype: A Pilot Study using targeted therapy in NSCLC. *Sci. Rep.* **2016**, *6*, 33860.

3. Chong, Y.; Kim, J.H.; Lee, H.Y.; Ahn, Y.C.; Lee, K.S.; Ahn, M.J.; Kim, J.; Shim, Y.M.; Han, J.; Choi, Y.L. Quantitative CT variables enabling response prediction in neoadjuvant therapy with EGFR-TKIs: are they different from those in neoadjuvant concurrent chemoradiotherapy? *PLoS ONE* **2014**, *9*, e88598.
4. Niu, L.; Qian, M.; Yang, W.; Meng, L.; Xiao, Y.; Wong, K.K.; Abbott, D.; Liu, X.; Zheng, H. Surface roughness detection of arteries via texture analysis of ultrasound images for early diagnosis of atherosclerosis. *PLoS ONE* **2013**, *8*, e76880.
5. Davnall, F.; Yip, C.S.; Ljungqvist, G.; Selmi, M.; Ng, F.; Sanghera, B.; Ganeshan, B.; Miles, K.A.; Cook, G.J.; Goh, V. Assessment of tumor heterogeneity: an emerging imaging tool for clinical practice? *Insights Imaging* **2012**, *3*, 573–589.
6. Lennon, F.E.; Cianci, G.C.; Cipriani, N.A.; Hensing, T.A.; Zhang, H.J.; Chen, C.T.; Murgu, S.D.; Vokes, E.E.; Vannier, M.W.; Salgia, R. Lung cancer-a fractal viewpoint. *Nat. Rev. Clin. Oncol.* **2015**, *12*, 664–675.
7. Wang, C.; Subashi, E.; Yin, F.F.; Chang, Z. Dynamic fractal signature dissimilarity analysis for therapeutic response assessment using dynamic contrast-enhanced MRI. *Med. Phys.* **2016**, *43*, 1335–1347.

Publisher's Note: MDPI stays neutral with regard to jurisdictional claims in published maps and institutional affiliations.

© 2020 by the authors. Licensee MDPI, Basel, Switzerland. This article is an open access article distributed under the terms and conditions of the Creative Commons Attribution (CC BY) license (http://creativecommons.org/licenses/by/4.0/).
